# ALK is a critical regulator of the MYC-signaling axis in ALK positive lung cancer

**DOI:** 10.18632/oncotarget.24260

**Published:** 2018-01-16

**Authors:** Amanda B. Pilling, Jihye Kim, Adriana Estrada-Bernal, Qiong Zhou, Anh T. Le, Katherine R. Singleton, Lynn E. Heasley, Aik Choon Tan, James DeGregori, Robert C. Doebele

**Affiliations:** ^1^ University of Colorado Cancer Center, Aurora, CO, USA; ^2^ Henry Ford Cancer Institute, Detroit, MI, USA

**Keywords:** ALK, ROS1, NSCLC, MYC, synthetic lethality

## Abstract

A subset of lung cancers is dependent on the anaplastic lymphoma kinase (*ALK*) oncogene for survival, a mechanism that is exploited by the use of the ALK inhibitor crizotinib. Despite exceptional initial tumor responses to ALK inhibition by crizotinib, durable clinical response is limited and the emergence of drug resistance occurs. Furthermore, intrinsic resistance is frequently observed, where patients fail to respond initially to ALK-inhibitor therapy. These events demonstrate the underlying complexity of a molecularly-defined oncogene-driven cancer and highlights the need to identify compensating survival pathways. Using a loss-of-function whole genome short-hairpin (shRNA) screen, we identified MYCBP as a determinant of response to crizotinib, implicating the MYC signaling axis in resistance to crizotinib-treated *ALK+* NSCLC. Further analysis reveals that ALK regulates transcriptional expression of *MYC* and activates c-MYC transactivation of c-MYC target genes. Inhibition of *MYC* by RNAi or small molecules sensitizes *ALK+* cells to crizotinib. Taken together, our findings demonstrate a dual oncogene mechanism, where ALK positively regulates the MYC signaling axis, providing an additional oncogene target whose inhibition may prevent or overcome resistance.

## INTRODUCTION

Non-small cell lung cancer (NSCLC) accounts for 85% of all lung cancer cases [1]. Molecular characterization of NSCLC has identified dominant molecular pathways that drive tumorigenesis, expanding treatment options to include targeted therapies that have contributed to improved patient outcome [2–4]. The gene rearrangement between the anaplastic lymphoma kinase (*ALK*) gene and echinoderm microtubule-associated protein-like 4 (*EML4*) gene resulting in the *EML4-ALK* gene fusion is the most common ALK fusion in lung cancer and are present in approximately 3-8% of NSCLC tumors, resulting in a constitutively active protein kinase that is essential for transformation [5, 6]. *EML4-ALK* transgenic mouse models have shown that ALK is required for growth and proliferation in cells and inhibition of ALK leads to growth inhibition and apoptosis [7]. In this regard, targeted inhibitors of ALK have been developed and used as an effective therapeutic strategy. Crizotinib, a kinase inhibitor with specificity for ALK, ROS1, and MET, was the first ALK inhibitor evaluated for clinical use and received accelerated FDA approval for treatment of *ALK* rearranged NSCLC based on an overall response rate of 57% in these patients [2, 8]. Despite this initial success, durable clinical response to crizotinib is limited by inevitable development of drug resistance through various known and unknown mechanisms of resistance. These include secondary mutations in the kinase domain*, ALK* gene copy number gain or gene amplification, and activation of alternative signaling pathways [9, 10]. Although more potent ALK inhibitors have been FDA approved, such as ceritinib, alectinib, and brigatinib, drug resistance still ultimately develops following treatment [11–14].

One approach to minimize drug resistance is to identify additional critical cellular pathways beyond the dominant oncogene and develop strategies targeting these vulnerabilities. Since there are numerous potential signaling mechanisms that may cooperate with the dominant oncogene, the use of functional genetic screens provides a powerful tool to explore mechanisms of drug resistance in preclinical cancer models. We describe here a genome-wide short hairpin RNA (shRNA) loss-of-function genetic screen to identify genes whose suppression can confer sensitivity to crizotinib in *ALK*+ NSCLC lines. We identified c-MYC binding protein (MYCBP) as a determinant of crizotinib sensitivity in *ALK+* cell lines. This is of significance since MYCBP has been shown to interact with and regulate oncogenic c-MYC transcriptional activity through promoting enhanced E-box recognition and transcription of c-MYC target genes [15]. In this report, we identified the MYC signaling network as critical for crizotinib sensitivity and that MYC activity is regulated in an ALK-dependent manner in *ALK* rearranged NSCLC.

## RESULTS

### RNAi-based screen identifies MYCBP as synthetic lethal with crizotinib in *ALK*+ NSCLC

**Figure 1:**
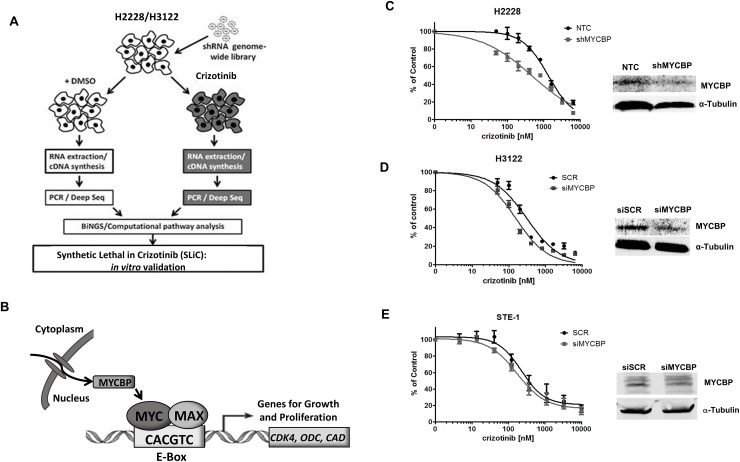
RNAi-based screen identifies MYCBP as synthetic lethal in *ALK+* NSCLC cell lines **A.** Schematic overview of genetic screen and data analysis workflow. **B.** Schematic representation of c-MYC and MYCBP interaction. *MYC* encodes the transcription factor c-MYC which heterodimerizes with Max and promotes transcription of genes involved in growth and proliferation. The mechanisms through which MYC mediates its broad range of biological effects are thought to include co-factors that can interact with c-MYC to regulate its transcriptional activity. MYCBP is one such co-factor that interacts with MYC through binding to its N-terminal domain to promote E-box dependent transcription of c-MYC target genes. **C.** H2228 cells expressing either non-gene targeting control (NTC) or shRNA targeting MYCBP (shMYCBP) were treated with crizotinib for 72 hours and proliferation was measured by MTS assay. Western blot demonstrates knockdown efficiency of MYCBP. H3122 **D.** or STE-1 **E.** cells expressing either scrambled siRNA control (siSCR) or SmartPool siRNA targeting MYCBP (siMYCBP) were treated with crizotinib for 72 hours and proliferation was measured by MTS assay. Knockdown efficiency was determined by Western blot.

In order to identify genes and pathways whose inhibition synergizes with crizotinib to induce cell death in *ALK* rearranged non-small cell lung cancer, we performed a genome-wide RNAi-based synthetic lethal screen in two *ALK+* NSCLC adenocarcinoma cell lines, H2228 and H3122 (Figure 1A). The screen used a lentiviral shRNA library carrying 3-5 target sequences for approximately 50,000 human gene transcripts. This library was transduced into H2228 and H3122 cells. After a selection period, transduced cells were divided into two groups and treated with either vehicle or crizotinib for 72h hours. shRNA sequences were amplified, sequenced, mapped to gene transcripts and analyzed for statistically significant changes between crizotinib and vehicle treated groups as described in Material and Methods.

Synthetic lethality was determined by identifying shRNAs that are underrepresented in the crizotinib treatment group as target genes whose silencing sensitized cells to crizotinib. To reduce false positives and to identify those genes that were indeed synthetic lethal hits, we considered genes with Z-transformed *p*-values (*P(wZP))* of <0.05 as synthetic lethal hits (325 and 226 genes for H2228 and H3122; Supplementary Table 1A and 1B, respectively) as previously described [16]. Based on this criteria, a total of 12 genes were significant hits in both H2228 and H3122 (Table 1). We have termed these genes as Synthetic Lethal in Crizotinib (SLiC) hits.

**Table 1:** Common synthetic lethal in crizotinib (SLiC) hits in H2228 and H3122

GENE SYMBOL	GENE NAME
CCDC59	Coiled-coil domain containing 59
SLC29A3	Solute carrier family 29 (nucleoside transporters), member 3
MCFD2	Multiple coagulation factor deficiency 2
RPL28	Ribosomal protein 28
PSMD1	Proteasome 26S subunit, non-ATPase 1
MYCBP	c-MYC binding protein
IKBKB	Inhibitor of nuclear factor kappa B kinase subunit Beta
RRM1	Ribonucleotide reductase subunit M1
GNB1	Guanine nucleotide binding protein subunit Beta 1
SSX2IP	Synovial sarcoma X breakpoint 2 interacting protein
PHC1	Polyhomeotic homolog 1
M6PR	Mannose 6-phophate receptor

Among the genes identified as SLiCs in both H2228 and H3122 *ALK+* cell lines was c-MYC binding protein (MYCBP), demonstrating post-analysis statistical significance determined by a weighted Z-transformed p-value and E-value. MYCBP encodes a small binding protein that functions as a c-MYC co-factor through binding to the N-terminal domain of c-MYC and stimulating E-box dependent transcriptional activation (Figure 1B) [15, 17]. Thus, identification of MYCBP in our synthetic lethal genetic screen suggests that the MYC signaling axis may be critical in *ALK+* NSCLC.

To validate MYCBP as synthetic lethal gene in our *ALK+* NSCLC cell lines, we used shRNAs targeting MYCBP whose shRNA sequences are distinct from those used in the initial screen. Vector shRNA targeting MYCBP and non-gene targeting negative control (NTC) were introduced into the *EML4-ALK* cell line H2228 to generate stable gene knockdown and negative control lines, respectively (Figure 1C). Knockdown of MYCBP in H2228 demonstrated a 3-fold increase in sensitivity to crizotinib. Stable knockdown of MYCBP was unsuccessful in the H3122 cell line, therefore validation of synthetic lethal inhibition was demonstrated in H3122 cells using a transient siRNA approach to silencing MYCBP (Figure 1D). MYCBP gene silencing decreased cell proliferation by 2-fold in response to crizotinib as compared to scrambled siRNA control (SCR). MYCBP gene silencing was performed in an additional *ALK*+ cell line, STE-1 (Figure 1E). Despite minimal knockdown efficiency, we still observe a modest increase in crizotinib sensitivity in this cell line. Finally, to demonstrate these effects were specific in ALK-activated NSCLC, MYCBP was effectively silenced in the EGFR mutant cell line HCC827 and treated with crizotinib (Supplementary Figure 1). Crizotinib had no effect on proliferation in this cell line and this remained unchanged upon MYCBP knockdown. Overall these results validate *MYCBP* as a critical gene whose inhibition increases sensitivity to crizotinib in *ALK+* NSCLC cell lines.

### MYC is regulated in an ALK-dependent manner in *ALK*+ NSCLC cells

**Figure 2:**
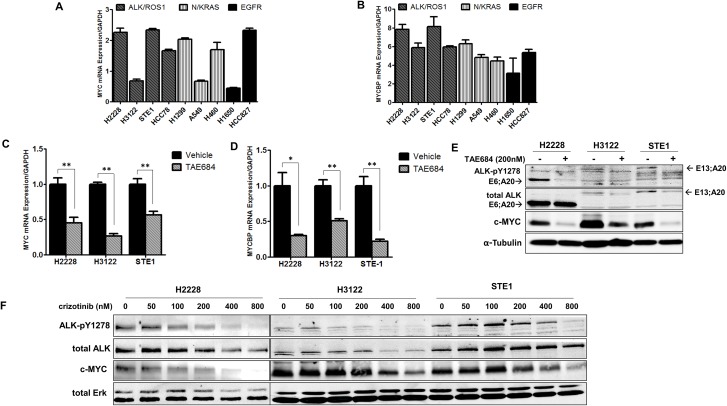
Regulation of MYC and *MYCBP* expression is ALK-dependent in NSCLC cells **A.** and **B.** Quantitative real-time PCR (qRT-PCR) analysis of *MYC* or *MYCBP* in NSCLC cells. NSCLC cell line panel includes *ALK+* cell lines (H2228, H3122, STE-1), a *ROS1* fusion line (HCC78), two *KRAS* cell lines (A549; G12S, H460; Q61H) and *NRAS* (H1299; codon 61), two mutant *EGFR* cell lines (H1650, HCC827; del 19). **C.** and **D.** Quantitative real-time PCR analysis of *MYC* or *MYCBP* in *ALK+* cell lines in response to 24 hour treatment with 200nM TAE684. (**P < 0.01, *P < 0.05). **E.** Western blot analysis of H2228, H3122 and STE-1 cells after 24 hour treatment with 200nM TAE684. **F.** Western blot analysis of *ALK+* cells treated for 24 hours with the indicated dose of crizotinib.

MYCBP can regulate the oncogenic transcriptional activity of MYC, which is frequently dysregulated in many human malignancies [18–20]. Furthermore, several studies have shown that aberrant ALK signaling in neuroblastoma cells regulates MYC and MYCN transcriptional initiation and protein stability [21, 22]. To determine if differential expression of MYC or MYCBP was present in *ALK+* NSCLC compared to other mutant-driver lung cancer subtypes, we measured basal mRNA and protein expression of MYC and MYCBP in a panel of cell lines that included three *ALK+* NSCLC cell lines (H3122, H2228, and STE-1), a *ROS1* gene fusion cell line (HCC78), two mutant *EGFR* cell lines (H1650 and HCC827) and two *KRAS* cell lines (A549 and H460) and *NRAS* (H1299) NSCLC cell lines (Figure 2A, 2B and Supplementary Figure 2). *MYC* mRNA expression at baseline is variable across all cell lines, reflecting the ubiquitous nature of MYC, and suggesting different mechanisms of regulation in a resting state. Analysis of *MYCBP* expression in this cell line panel showed that the *ALK+* cell lines showed a trend towards greater transcript levels of *MYCBP* compared with the other genomic subtypes.

To further investigate MYC regulation in *ALK+* NSCLC, we examined the expression of MYC and MYCBP in response to pharmacologic ALK inhibition in *ALK+* NSCLC cell lines. To eliminate any potential off-target effects, we used TAE684, a potent, highly specific ALK inhibitor [23], as a complementary inhibitor tool for these studies. Treatment with TAE684 reduced *MYC* transcript levels in each of the *ALK+* cell lines tested (Figure 2C). We also examined expression of *MYCBP* in response to ALK inhibition, as it has been demonstrated that *MYCBP* expression can be regulated indirectly through *MYC* [24, 25]. Similar to that of *MYC,* we observed decreased transcript levels of *MYCBP* in response to TAE684 (Figure 2D). We observe similar results in response to crizotinib (Supplementary Figure 3). Immunoblot analysis revealed loss of c-MYC protein expression in the three *ALK*+ cell lines tested in response to TAE684 treatment (Figure 2E). To verify inhibition of the auto-activated ALK kinase domain, an antibody recognizing phosphorylated tyrosines 1278/1282/1283 was employed. Analysis of several NSCLC ROS1 fusion cell lines also demonstrated loss of c-MYC protein in response to TAE684 (Supplementary Figure 4). This is important since ALK and ROS1 are evolutionary conserved kinases and share 77% amino acid identity within the ATP-binding sites, permitting parallel therapeutic approaches against the two kinases [26]. Additionally, we demonstrate reduction of c-MYC protein in response to a dose range of crizotinib (Figure 2F). Furthermore, the observed loss of MYC protein in response to ALK inhibitor is ALK-specific, as we do not see concomitant reduction in MYC protein levels in KRAS or EGFR mutant cell lines treated with an ALK inhibitor (Supplementary Figure 5).

### ALK regulates MYC protein stabilization and transcription initiation in *ALK*+ NSCLC

**Figure 3:**
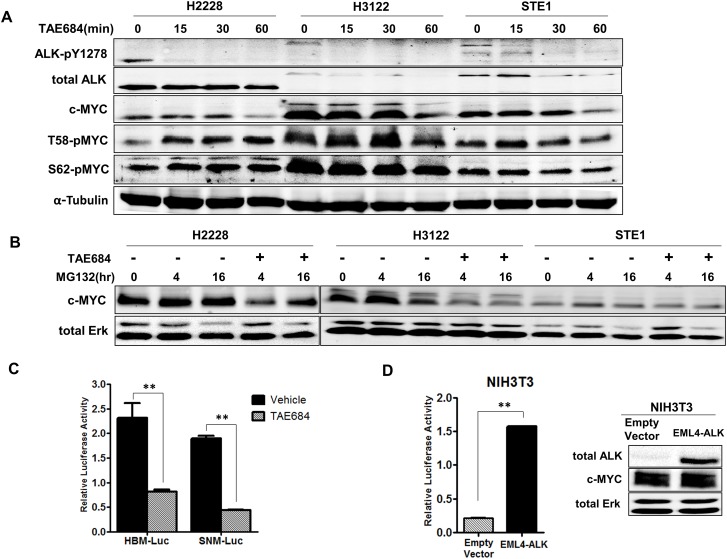

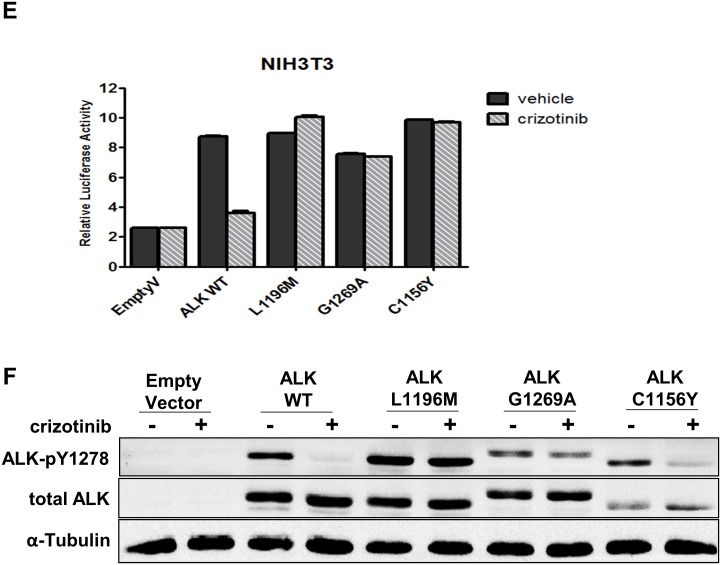
ALK regulates the *MYC* promoter and minimally regulates c-MYC protein stabilization in *ALK*+ NSCLC cells **A.** Western blot analysis of c-MYC phosphorylation sites in *ALK+* cell lines treated with 200nM TAE684 for up to 60 minutes. **B.** Western blot analysis of *ALK+* cell lines treated with proteasome inhibitor MG132 alone or in combination with TAE684 for 0, 4, or 16 hours. **C.** Luciferase activity measuring *MYC* promoter activity in H3122 cells expressing HBM-Luc or SNM-Luc promoter construct treated with 200nM TAE684. (**P < 0.01). **D.** Luciferase activity measuring *MYC* promoter activity in NIH3T3 cells co-transfected with *EML4-ALK* or pCDH-Empty Vector and HBM-Luc constructs. Western blot showing expression of ALK in *EML4-ALK*-expressing NIH3T3 cells. (**P < 0.01). **E.** Luciferase activity measuring *MYC* promoter activity in NIH3T3 cells co-transfected with pCDH-empty vector or *EML4-ALK* wild-type (WT) or the cDNA encoding L1196M, G1269A or C1156Y mutations and the HBM-Luc *MYC* reporter construct. **F.** Western blot analysis of NIH3T3 lysates expressing the indicated cDNA vector treated with crizotinib.

Regulation of c-MYC can occur through post-translational modifications at MYC-homology Box 1 (MB1) within the N-terminal domain [27]. Phosphorylation events at serine 62 (S62) and threonine 58 (T58) can regulate c-MYC protein stability, where phosphorylation at S62 and T58 together promotes MYC protein stability, but phosphorylation of T58 alone leads to ubiquitination and degradation [28]. To explore c-MYC protein stability in *ALK+* NSCLC we analyzed the phosphorylation of these sites in our three *ALK+* cell lines in response to TAE684 (Figure 3A). Overall, we observe sustained S62 phosphorylation, indicating c-MYC protein stability is not significantly altered through loss of ALK kinase signaling. However, we observe increased phosphorylation at T58 in H2228 cells in response to TAE684, indicating c-MYC protein stability may be regulated by ALK in this cell line. The levels of S62 (which promotes c-MYC protein stability) remain unchanged suggesting balanced c-MYC protein levels. Minor changes in T58 and S62 phosphorylation are observed in H3122 and STE-1 cells, suggesting ALK may in part play a role in mediating c-MYC protein stability in these cell lines, but does not fully explain significant reduction in total c-MYC protein levels.

To further investigate regulation of c-MYC protein levels in *ALK+* cells, we examined c-MYC protein turnover by treating cells with crizotinib and the proteasome inhibitor MG132 (Figure 3B). Overall, we observe increased c-MYC protein levels within 4 hours of MG132 treatment alone, indicating protein is accumulating and not undergoing degradation. In H3122 and STE-1 cells, pre-treatment with ALK inhibitor TAE684 demonstrates reduced c-MYC expression that is not rescued by addition of the proteasome inhibitor, indicating ALK-mediated regulation of MYC is not at the protein level. In the H2228 cell line, we observed some recovery of c-MYC protein after 16 hour treatment with TAE684 in presence of MG132. Since H2228 also demonstrated increased T58 phosphorylation in response to ALK inhibition, this indicates that ALK may be involved in regulating c-MYC protein levels in this cell line.

We next investigated if ALK is involved in transcriptional regulation of *MYC*, as demonstrated in other ALK-activated tumor types [22, 29, 30]. In order to investigate the effect of ALK activity on initiation of transcription of *MYC,* we employed promoter-reporter luciferase technology. We used two *MYC* reporter constructs, SNM-Luc and HBM-Luc, which contain the required P1 and P2 *MYC* transcription start sites and engineered with a luciferase reporter (Supplementary Figure 6). HBM-Luc is the full-length promoter sequence where SNM-Luc is a truncated reporter, created by terminal deletion of a 502-bp fragment from the 5’ end of the HBM-Luc construct while still containing both P1 and P2 transcription start sites [31, 32]. We transiently transfected these constructs into H3122 cells and measured the luciferase activity after treatment with TAE684 (Figure 3C). As demonstrated, both the HBM-Luc and SNM-Luc promoter constructs were responsive to ALK inhibition indicating that only the minimal sequences for correct transcription of *MYC* are required for ALK to regulate *MYC* promoter activity. Similarly, we performed these experiments with a dose range of crizotinib and observe reduced promoter activity within the known IC_50_ range of H3122 (~150nM) (Supplementary Figure 7). Additionally, in the *ALK+* NSCLC cell line STE-1, we observe reduced MYC-promoter activity in response to TAE684, in both reporter assays (Supplementary Figure 8).

To determine if the activation of the *MYC* promoter is ALK-specific, we co-transfected NIH3T3 cells with *EML4-ALK* cDNA and HBM-Luc *MYC* reporter construct and measured luciferase activity compared to cells co-transfected with pCDH-empty vector cDNA and HBM-Luc *MYC* reporter construct (Figure 3D). The *MYC* promoter activity in the cells co-transfected with the *EML4-ALK* vector was 5-fold higher compared to empty vector indicating that ALK is indeed activating the promoter activity of *MYC*.

To confirm ALK activity increases *MYC* promoter activity and the observed decrease in luciferase activity with ALK inhibitor is not an off-target (ALK non-specific) pharmacological effect, we utilized *EML4-ALK* cDNA vectors encoding mutations in the ALK kinase domain that have previously been determined to confer resistance to crizotinib [9]. We co-transfected NIH3T3 cells with cDNA vectors containing pCDH-empty vector or wild-type *EML4-ALK*, or the same cDNA encoding L1196M, G1269A or C1156Y mutations plus the HBM-Luc *MYC* reporter construct and measured luciferase activity in response to crizotinib (Figure 3E and 3F). The NIH3T3 cells expressing the *EML4-ALK* resistant mutations abrogated the impact of crizotinib treatment on *MYC* promoter activity as compared to those containing wild type *EML4-ALK,* conferring resistance in the context of *MYC* expression. Overall, these results implicate ALK in the transcriptional regulation of *MYC* in *ALK* rearranged NSCLC.

### ALK regulates transcriptional activity of c-MYC in *ALK*+ NSCLC

**Figure 4:**
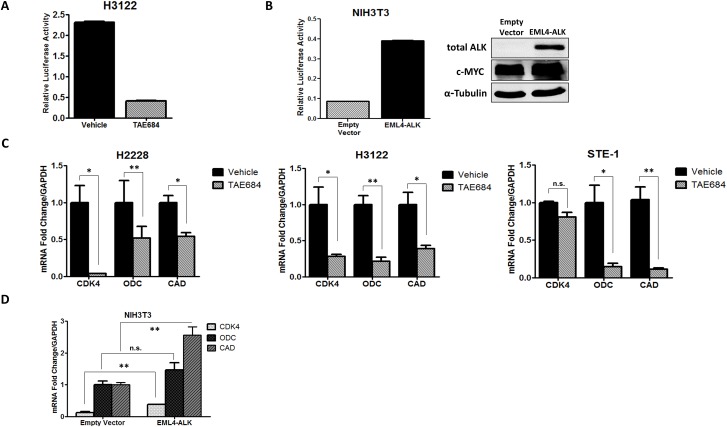
ALK regulates transcriptional activity of c-MYC in *ALK*+ NSCLC cells **A.** Luciferase activity measuring c-MYC target gene promoter activity in H3122 cells expressing the MBS-Luc reporter construct upon treatment with 200nM TAE684. **B.** Luciferase activity measuring c-MYC target gene promoter activity in NIH3T3 cells co-transfected with *EML4-ALK* or pCDH-Empty Vector and MBS-Luc constructs. **C.** Quantitative real-time PCR analysis of c-MYC target genes cyclin-dependent kinase (CDK4), ornithine decarboxylase (ODC), carbamoyl phosphate synthase-aspartate transcarbamylase-dihydroorotase (CAD) in *ALK+* cell lines treated with 200nM TAE684. **D.** Quantitative real-time PCR analysis of c-MYC target genes CDK4, ODC, and CAD in NIH3T3 cells co-transfected with *EML4-ALK* or pCDH-Empty Vector. (**P < 0.01, *P < 0.05).

We sought to determine if transcriptional activity of c-MYC was mediated in an ALK-dependent manner. We used a luciferase promoter-reporter construct, MBS-Luc, containing four c-MYC E-box consensus sequence binding sites (MBS) upstream of a promoter of the known c-MYC target gene, CDK4 (Supplementary Figure 9) [33]. We transfected H3122 cells with the MBS-Luc reporter construct and measured luciferase activity after treatment with 150nM of crizotinib (Figure 4A). In response to crizotinib, we observe over a 5-fold reduction in luciferase activity, indicating ALK is critical in mediating c-MYC transcriptional activation. Next, we tested whether ALK could induce c-MYC transcriptional activity in a non-tumor cell line ectopically expressing *ALK*. We employed NIH3T3 cells and co-transfected them with wild-type *EML4-ALK* or pCDH empty vector in addition to the MBS-Luc reporter construct (Figure 4B). Here we observe a nearly 8-fold induction of c-MYC transcriptional activity in ALK-expressing cells demonstrating that ALK induces c-MYC transcriptional activity. To further assess ALK-dependent regulation of c-MYC activity, we examined mRNA expression of known c-MYC target genes in response to ALK inhibitor TAE684 (Figure 4C). The c-MYC target genes analyzed include cyclin-dependent kinase (CDK4), ornithine decarboxylase (ODC), and carbamoyl phosphate synthase-aspartate transcarbamylase-dihydroorotase (CAD), all of which are implicated in oncogenic signaling. After treatment with TAE684 we observe decreased expression of each of the c-MYC target genes in all three *ALK*+ cell lines tested. To test the ability of ALK to induce c-MYC target gene expression, we transfected NIH3T3 cells with wild-type *EML4-ALK* cDNA or empty vector and demonstrate that expression of *ALK* in the NIH3T3 cells induces expression of these oncogenic c-MYC target genes (Figure 4D). Overall, these results demonstrate that ALK mediates regulation of c-MYC transcriptional activities and induces expression of c-MYC target genes, therefore establishing ALK as a key activator of oncogenic MYC signaling in *ALK+* NSCLC.

### Inhibition of MYC as a potential treatment strategy in *ALK*+ NSCLC

**Figure 5:**
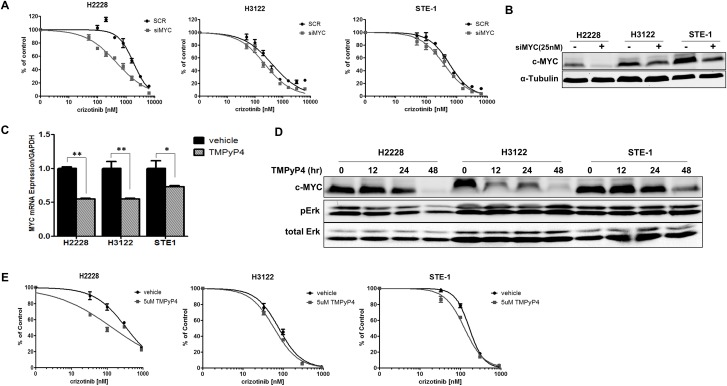
Inhibition of *MYC* sensitizes *ALK*+ NSCLC cells to crizotinib **A.** H2228, H3122 or STE-1 cells expressing either scrambled siRNA control (siSCR) or SmartPool siRNA targeting *MYC* (siMYC) were treated with crizotinib for 72 hours and proliferation was measured by MTS assay. **B.** Western blot demonstrates knockdown efficiency of *MYC*. **C.** Quantitative real-time PCR analysis of *MYC* in *ALK*+ cells after treatment with 5μM TMPyP4 for 48 hours. (**P < 0.01, *P < 0.05). **D.** Western blot analysis demonstrating c-MYC protein expression after treatment with 5μM TMPyP4 for up to 48 hours. **E.** *ALK*+ cells were treated with crizotinib in combination with either vehicle or 5μM TMPyP4 for 72 hours and proliferation was measured by MTS assay.

Aberrant expression of MYC is a common feature in many human malignancies and may be critical in *ALK+* NSCLC. MYC therefore represents an attractive therapeutic target. To observe the effects of *MYC* loss in *ALK+* NSCLC, we employed RNAi technology to silence *MYC* in *ALK*+ cell lines and measured the proliferation in response to crizotinib (Figure 5A). We observed increased crizotinib sensitivity with *MYC* silencing (siMYC). Interestingly, this appears to be dose-dependent with the level of knockdown observed across the cell lines tested (Figure 5B). In order to correlate the observed effects of RNA silencing to a pharmacologically relevant target, we used TMPyP4, a small molecule capable of stabilizing a specific G-quadruplex within the promoter of *MYC* and thus inhibiting its expression [34]. We demonstrate reduced *MYC* mRNA expression and loss of c-MYC protein expression upon treatment with TMPyP4 in the *ALK+* NSCLC cells (Figure 5C and 5D). In order to see the effect of combined ALK inhibition with TMPyP4, we treated *ALK+* cell lines with increasing dose of crizotinib in the presence of 5μM of the TMPyP4 compound (Figure 5E). We observe increased sensitivity to crizotinib in the presence of the TMPyP4 MYC inhibitor, similar to the *MYC* RNAi analysis. Taken together, these results demonstrate reduction of *MYC* increases sensitivity to ALK inhibition in *ALK+* NSCLC and provides rationale for dual inhibition in the therapeutic setting.

## DISCUSSION

The profound initial responses observed with the use of targeted therapies, such as ALK inhibitors, in the treatment of oncogene-driven lung adenocarcinoma has demonstrated the value of identifying the dominant signaling pathways driving the cancer. However, complete responses are not durable and resistance inevitably emerges. Furthermore, some *ALK* positive lung cancer patients do not exhibit significant tumor shrinkage with ALK-inhibitor treatment, suggesting refractory residual disease may result from compensatory signaling pathways [11, 35]. Recent work by our lab demonstrated a critical role for EGFR signaling in *ALK+* cancer cells [36]. By understanding the additional signaling requirements of these oncogene-positive cancer cells, we can begin to identify compensatory survival pathways that mediate the intrinsic and acquired resistance and improve treatments strategies.

Alternative or effector signaling pathways are emerging as mechanisms of resistance in the era of oncogene-targeted therapy. As reported recently, *ALK+* lung cancer cells demonstrate a dependence on MAPK signaling for growth and survival and as a mechanism of ALK inhibitor resistance [37]. In order to identify genes (and pathways) whose suppression can confer sensitivity to crizotinib, we used a loss-of-function genetic screen in *ALK* rearranged lung cancer cell lines. The results from the screen uncovered *MYCBP* as a target in sensitizing *ALK+* NSCLC to crizotinib, implicating the MYC signaling axis as critical in this tumor type. *MYC* encodes the oncogenic transcription factor c-MYC, which heterodimerizes with Max and promotes transcription of genes required for growth and proliferation. The mechanisms through which c-MYC mediates its broad range of biological effects are thought to include co-factors that can interact with c-MYC to regulate its transcriptional activity. MYCBP is one such co-factor that interacts with c-MYC through binding to the N-terminal transactivation domain and promotes E-box dependent transcription of c-MYC target genes [15]. This co-factor regulation of c-MYC may be critical in *ALK+* NSCLC survival as we demonstrate that RNAi-mediated loss of *MYCBP* increases sensitivity to ALK-inhibitor crizotinib in *ALK+* NSCLC cell lines, therefore validating our findings from the synthetic lethal gene screen. It is notable that another hit in this synthetic lethal screen, EGFR, has recently been validated by our lab [36].

The association between activating *ALK* mutations and MYC or MYCN is observed in other malignancies including neuroblastoma and anaplastic large cell lymphoma (ALCL). A connection between ALK and MYC has been observed in neuroblastoma, where ALK gain-of-function mutations are more frequent in *MYCN* amplified tumors and activation of full length ALK increases mRNA, protein expression, and transformation potential of MYCN in neuroblastoma cell lines [21, 22]. In ALCL, activation of ALK through a conditionally dimerized construct results in increased c-MYC protein and *MYC* mRNA expression [38]. Furthermore, it was demonstrated that NPM-ALK-induced expression of *MYC* is mediated through interferon regulatory factor (IRF4), a transcription factor shown to bind the *MYC* promoter and induce *MYC* expression [29]. In neuroblastoma, ALK activity has been shown to promote transcription of *MYCN* through phosphorylation of ERK5, a member of the MAPK family, in a PI3K-dependant manner thus integrating ALK effector signaling and MYC regulation [22, 30].

In this present study, we observe decreased abundance of *MYC* mRNA and c-MYC protein expression in response to ALK inhibition implicating ALK in regulation of MYC in NSCLC. The loss of c-MYC protein expression was also observed in several ROS1 fusion cell lines in response to TAE684, consistent with highly homologous kinase domains between ALK and ROS1. Furthermore, we demonstrate regulation of *MYC* promoter activity by ALK in *ALK+* cell lines and non-tumor NIH3T3 ectopically expressing *EML4-ALK*. Additional analysis using mutant ALK kinase domain expression vectors with known crizotinib resistance activity in NIH3T3 cells, demonstrates abrogated responses to crizotinib in the ALK-mutants thus diminishing the effect of ALK inhibitor on c-Myc transcriptional activity. This finding not only demonstrates that ALK is necessary for *MYC* transcription in this model, but also points to a possible mechanism of oncogene co-operation in the resistance setting. Previous studies have demonstrated enhanced MYCN protein stability by constitutive activation of ALK [21]. In our study, we do not observe definitive regulation at the protein level as ALK inhibition fails to alter c-MYC phosphorylation status in H3122 and STE-1 cells and furthermore, cells pre-treated with ALK inhibitor and then exposed to proteasome inhibition exhibit loss of c-MYC expression indicating possible regulation at the level of transcription. However, based on the observation of modest increase in phosphorylation at T58 upon crizotinib treatment in H2228 cells, we cannot completely rule out protein stability as a mechanism of MYC regulation by ALK. Additionally, it is thought that regulation of protein stability by ALK effector signaling and ALK-mediated regulation of *MYC* transcription are not mutually exclusive [21, 30, 39]. MYC is a potent activator of oncogenic transcription programs and demonstrated to be required for tumor growth in multiple tumor types. In a retrospective analysis in lung adenocarcinomas, *c-MYC* copy number gain was an independent poor-prognostic factor for disease-free and overall survival, with a possible association with EGFR mutation status [40]. We examined the role of ALK in c-MYC transactivation of known target genes and find that c-MYC transactivation can be regulated by ALK. The mechanism through which ALK induces activation of c-MYC target genes is unknown. Our group recently performed analysis of circulating tumor DNA from *ALK+* patients collected prior to initiating treatment and at time of progression on ALK inhibitor. MYC amplification was observed in 2/42 ALK-inhibitor resistant patient samples suggesting MYC overexpression or amplification as a potential mechanism of resistance to ALK inhibitors [41].

Since MYC and ALK could be cooperative in oncogenic signaling and therapy resistance, we examined the effect of combination inhibition in *ALK+* NSCLC cells. RNAi-mediated silencing of MYC increased sensitivity to ALK inhibition. Additionally, use of a G-quadruplex inhibitor that inhibits transcription of *MYC* also resulted in increased sensitivity to ALK inhibition. These results indicate that the MYC axis is critical and has the potential to be therapeutically exploited in *ALK+* NSCLC. In summary, through a genome-wide RNAi screen we identified MYCBP as a potential target in sensitization to ALK kinase inhibition. We demonstrate that the MYC signaling axis is critical in *ALK+* NSCLC and is positively regulated by ALK providing a potential therapeutic opportunity for combination therapy.

## MATERIALS AND METHODS

### Cell lines and reagents

H2228, H3122, and HCC78 were a kind gift from Dr. John D. Minna (The University of Texas, Southwestern Medical Center, Dallas, TX) and STE-1 were a kind gift from Dr. Christine M. Lovly (Vanderbilt University School of Medicine, Nashville, TN). ROS1 fusion cell lines CUTO2 (*SDC4-ROS1*), CUTO23 and CUTO27 (*CD74-ROS1*), and CUTO28 (*TPM3-ROS1*) were generated at the University of Colorado from patient tumor samples following informed consent under an IRB-approved protocol. ALK and ROS1 fusion cell lines were grown in RPMI supplemented with 10% FBS. H1299, A549, H460, H1650, HCC827 were obtained from the University of Colorado Cancer Center Tissue Culture Core and cultured in RPMI supplemented with 5% FBS. NIH3T3 cells were obtained from American Type Culture collection and grown in Dulbecco’s Modified Eagle’s Media with 5% FBS. Crizotinib (PF-02341066) was obtained from Pfizer, Inc. NVP-TAE684 and MG132 were purchased from Selleck Chemicals. Antibodies used were ALK pY1278/1282/1283 (#3983), total ALK (#3791), ERK pT202/Y204 (#9191), total ERK (#9107), c-MYC (#5605), c-MYC pS62 (#13748) were purchased from Cell Signaling Technology. MYCBP (ab172444) c-MYC pT58 (28842) was purchased from Abcam, and alpha-tubulin (sc-8035), was purchased from Santa Cruz biotechnology.

### Proliferation assays

Proliferation was measured using the MTS CellTiter 96 Aqueous Proliferation Assay from Promega according to the manufacturer’s instructions. Briefly, cells were seeded into 96-well plates at a density to permit exponential growth throughout the length of the assay 24 hours before drug treatment, and proliferation was measured 72 hours after treatment. The absorbance at 490 nm was measured in 96-well plates using a Microplate Reader from Molecular Devices. The IC_50_ values were calculated using Prism v5.02 from GraphPad Software.

### Immunoblotting

Immunoblotting was conducted as previously described with minor modifications [9]. Briefly, cells were lysed in modified RIPA buffer supplemented with Halt Protease and Phosphatase Inhibitor Cocktail purchased from Thermo Scientific. Total protein was separated by SDS-PAGE, transferred to nitrocellulose, and stained with the indicated primary antibodies. Protein detection was achieved by imaging with an Odyssey Imager and Odyssey Version 3.0 image analysis software from LI-COR Biotechnology.

### Quantitative real-time PCR (qRT-PCR)

Total RNA (1ug) was reverse transcribed using High Capacity cDNA Reverse Transcription Kit from Life Technologies. 1uL of cDNA was PCR amplified in a 20uL reaction including TaqMan 2X Universal Master Mix and TaqMan gene expression probe/primer set for *MYC* (Hs00153408), *MYCBP* (Hs01894873), and *GAPDH* (Hs02758991) as an internal control for normalized gene expression. Samples were run in triplicate for a total of 3 separate experiments.

### Luciferase reporter assays

H3122, STE-1 or NIH3T3 cells were transfected with 5ug of promoter-driven firefly luciferase plasmid and 5ug TK-Renilla using TransIT-2020 reagent (Mirus, Madison, WI). After treatment incubation, cells were lysed and assayed following Dual-Luciferase Reporter Assay System instructions (Promega, Madison, WI). The HBM-Luc, SNM-Luc, and MBS-Luc promoter luciferase constructs were obtained from Addgene (Cambridge, MA). Lentivirus preparation

Lentivirus production was performed by transfecting viral packaging vectors pCMV-VSV-G and pΔ8.9 into 293T cells using TransIT-293 reagent (Mirus) as previously described [42]. For the genome-wide screen GeneNet Lentiviral Human 50K library was used (pS1H1-H1 Puro; Systems Biosciences). shRNA vectors used for validation were sourced from TRC (pLKO.1).

### shRNA synthetic lethal gene screen

The screen used a lentiviral shRNA library carrying 3-5 target sequences for approximately 50,000 human gene transcripts (SBI, Mountain View, CA). This library was transduced into H2228 and H3122 cells at a low MOI to allow for one gene knockdown per cell and selected in puromycin for two weeks to eliminate untransduced cells. This period of growth also allowed for elimination of shRNAs that target essential genes. The population of transduced cells was then divided into two groups for treatment with vehicle control or the ALK inhibitor crizotinib at an IC80 for both cell lines (H2228: 300nM, H3122: 150nM). Total RNA was isolated and reverse-transcribed, cDNA was amplified by nested PCR with addition of adapter sequences. Sequencing was performed on an Illumina Genome Analyzer and shRNAs were identified and the number of clusters for each shRNA sequence was quantified.

### Bioinformatics analysis

The Bioinformatics for Next Generation Sequencing! (BiNGS!) workflow was used to analyze and interpret the genome-wide synthetic lethal data [16]. A pre-processing step removed low quality and erroneous reads, typically non-barcoded sequences. Reads were then mapped to the shRNA reference library using Bowtie [43]. Additional filtering removed shRNAs mapping to sequences of unannotated genes, shRNAs where the median raw count was greater in the control than the maximum raw count in the treatment group if the shRNA is enriched in the treatment group, and vice versa (medC > maxT). A negative binomial was used to model the read count distribution in the data using edgeR [44]. Post-analysis determined the q-value of false discovery rate for multiple comparisons of the shRNAs and collapsed shRNAs to genes by combining q-values for all the shRNAs representing the same gene using weighted Z-transformation [45]. The associated weighted Z-transformed p-value (P(wZP)) is used to sort the gene list demonstrating differentially represented shRNAs, identifying synthetic lethal genes.

### Statistical analysis

Statistical significance was assessed by Student’s t-Test (two-tailed distribution, two-sample, unequal variance) and considered statistically significant with *P*-value <0.05, (**P < 0.01, *P < 0.05).

## SUPPLEMENTARY MATERIALS FIGURES AND TABLES






