# Supplementary material for: ALK is a critical regulator of the MYC-signaling axis in ALK positive lung cancer
Source: Oncotarget. 2018 Jan 16;9(10):8823–35. doi: 10.18632/oncotarget.24260 (PMC5823650; doi:10.18632/oncotarget.24260)
Supplement: Supplementary file 1 [file oncotarget-09-8823-s001.pdf]

ALK is a critical regulator of the MYC-signaling axis in ALK positive lung cancer

SUPPLEMENTARY MATERIAL

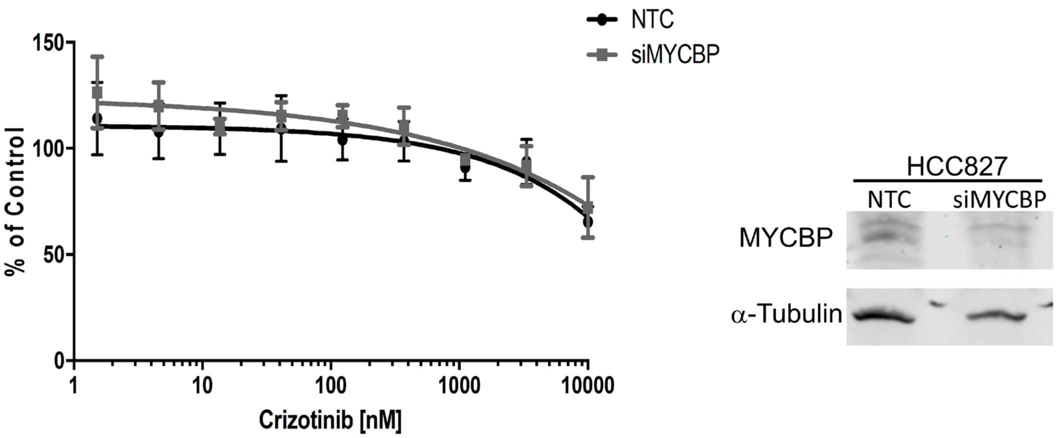

Supplementary Figure 1: HCC827 cells expressing either scrambled, non-targeting siRNA control (siNTC) or SmartPool siRNA targeting MYCBP (siMYCBP) were treated with crizotinib for 72 hours and proliferation was measured by MTS assay. MYCBP knockdown efficiency was determined by Western blot.

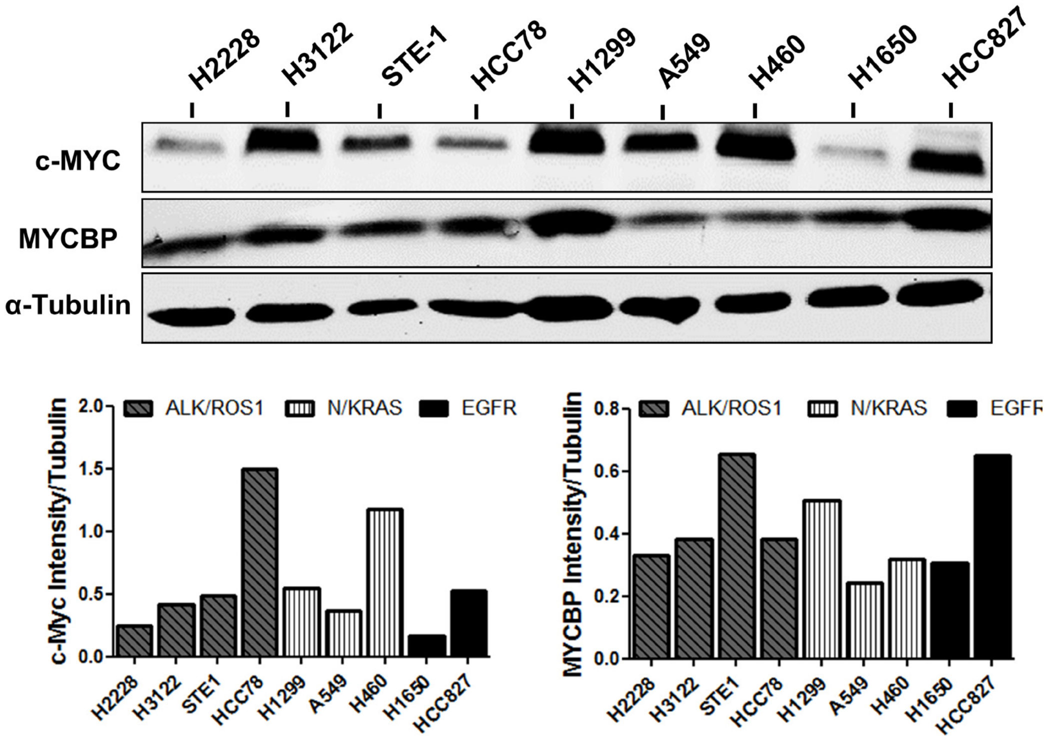

Supplementary Figure 2: Western blot analysis of c-MYC and MYCBP expression in a panel of NSCLC cells. Quantitation of integrated intensity of c-MYC and MYCBP expression normalized to α-tubulin.

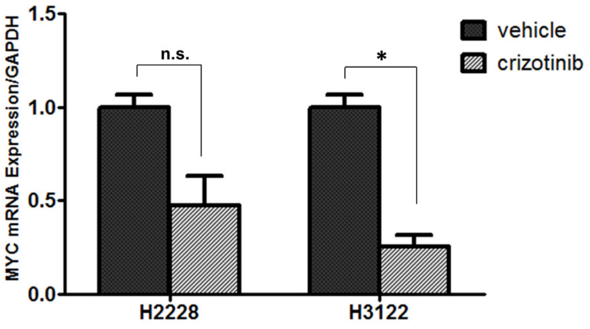

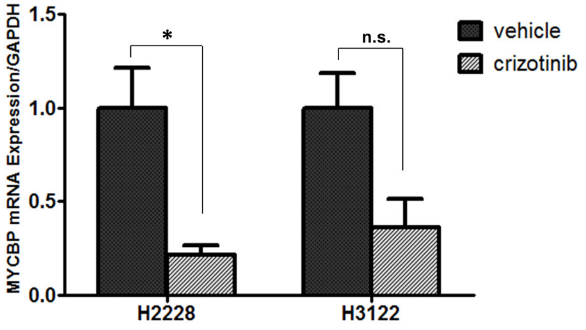

Supplementary Figure 3: Quantitative real-time PCR analysis of *MYC* or *MYCBP* in H2228 or H3122 cells after 24 hour treatment with 100nM or 500nM crizotinib, respectively.

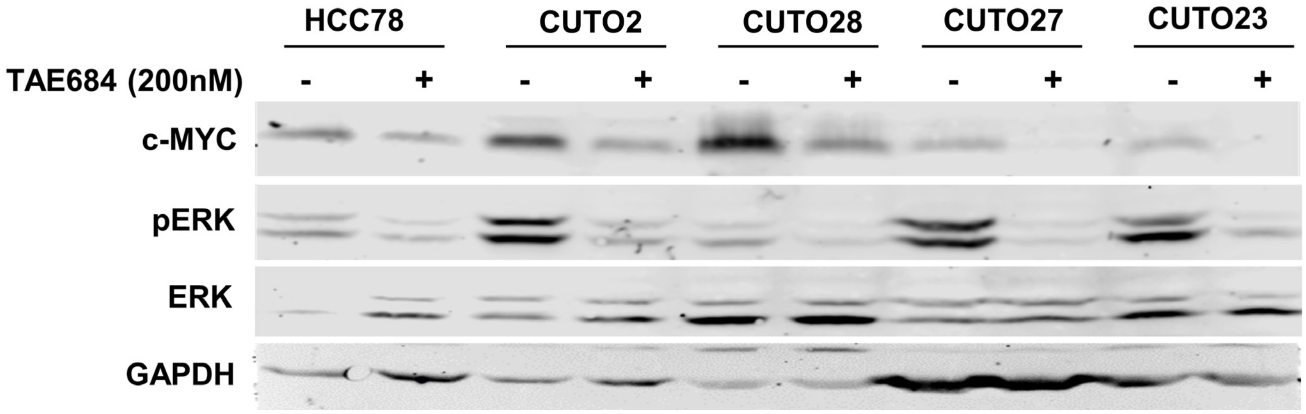

Supplementary Figure 4: Western blot analysis of c-MYC protein expression in ROS1 fusion cell lines HCC78 *(SLC34A2-ROS1)*, CUTO2 *(SDC4-ROS1)*, CUTO23 and CUTO27 *(CD74-ROS1)*, and CUTO28 *(TPM3-ROS1)* treated with 200nM TAE684 for 2h.

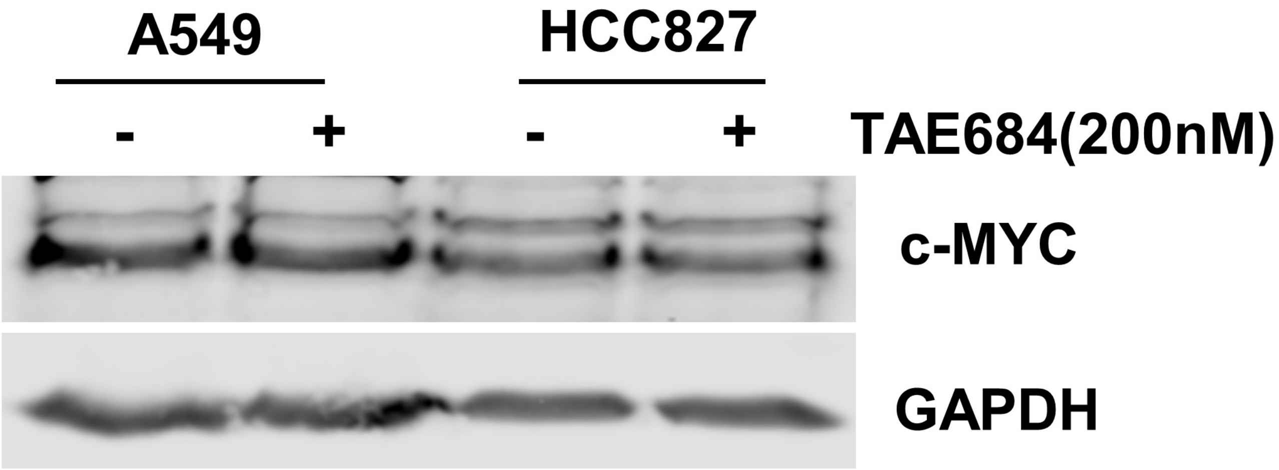

Supplementary Figure 5: Western blot analysis of c-MYC protein expression in cell lines A549 (*KRAS* mut) and HCC827 (*EGFR* mut) treated with 200nM TAE684 for 2h.

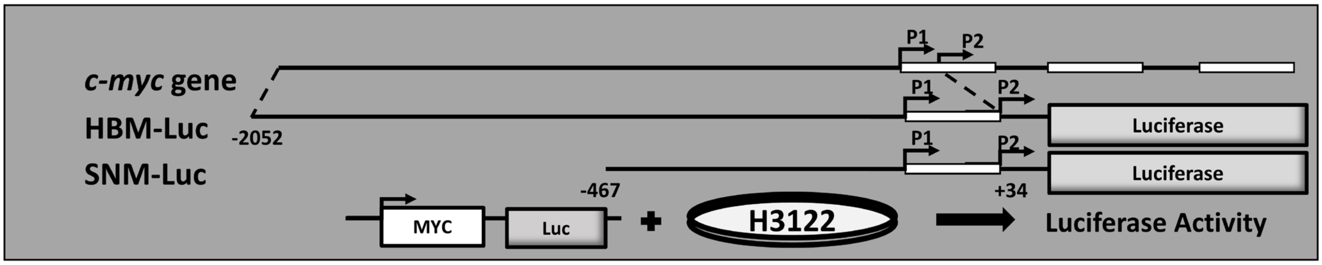

Supplementary Figure 6: In-line schematic of promoter reporter luciferase constructs and *MYC* gene. HBM-Luc: full length construct; SNM-Luc: minimal promoter.

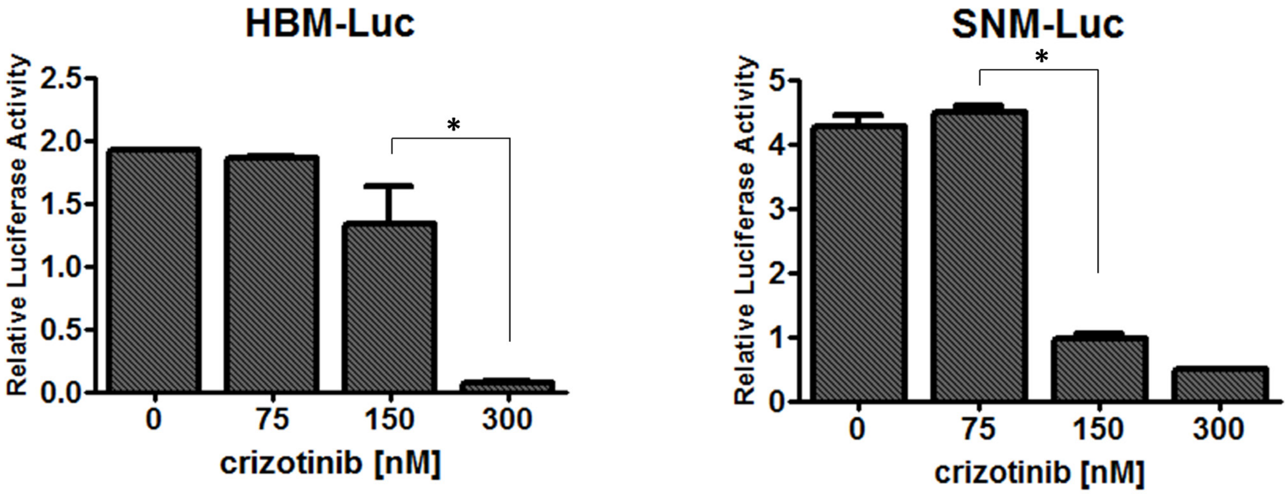

Supplementary Figure 7: Luciferase activity measuring *MYC* promoter activity in H3122 cells expressing HBM-Luc or SNM-Luc promoter construct upon treatment with crizotinib.

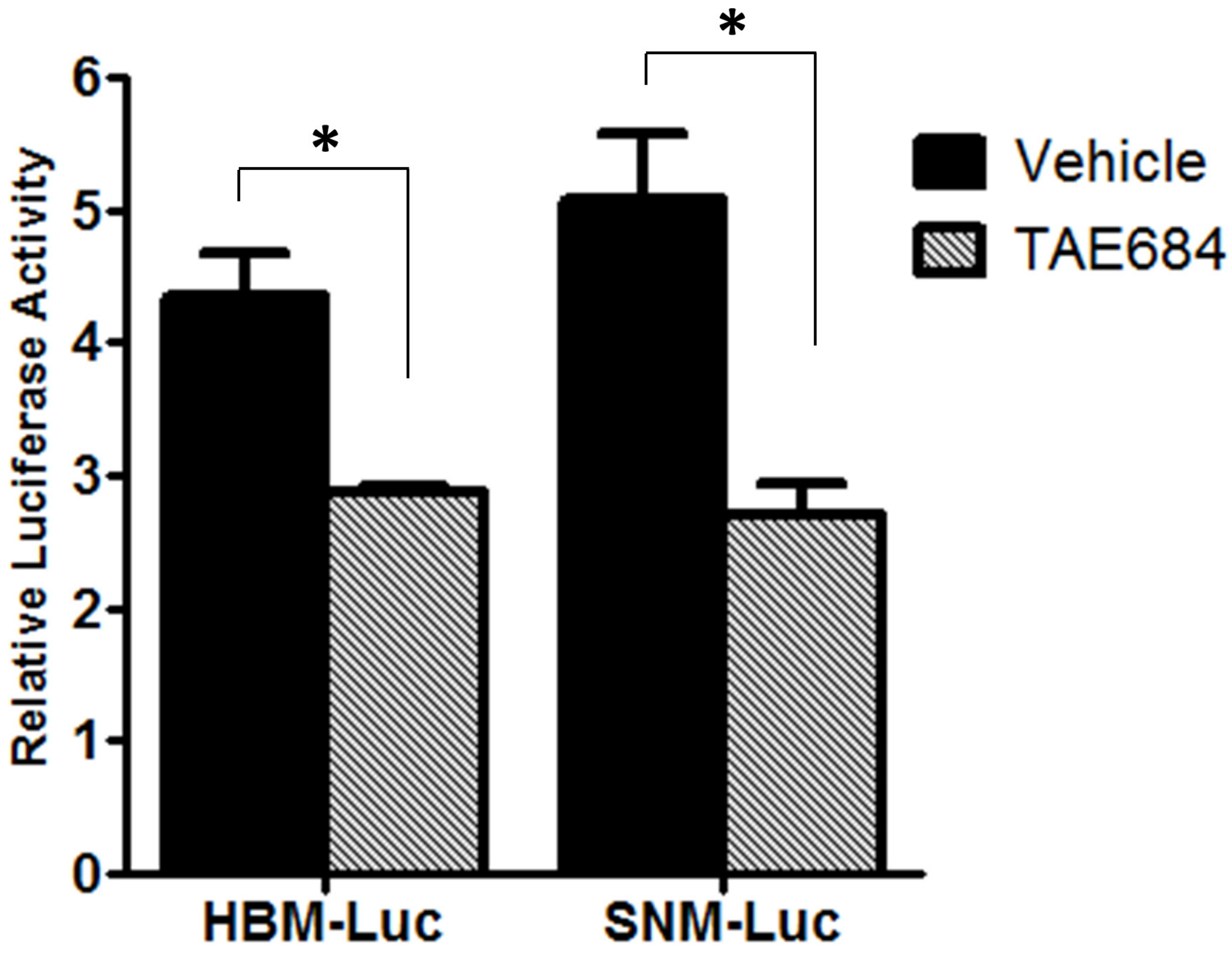

Supplementary Figure 8: Luciferase activity measuring *MYC* promoter activity in STE-1 cells expressing HBM-Luc or SNM-Luc promoter construct upon treatment with 200nM TAE684.

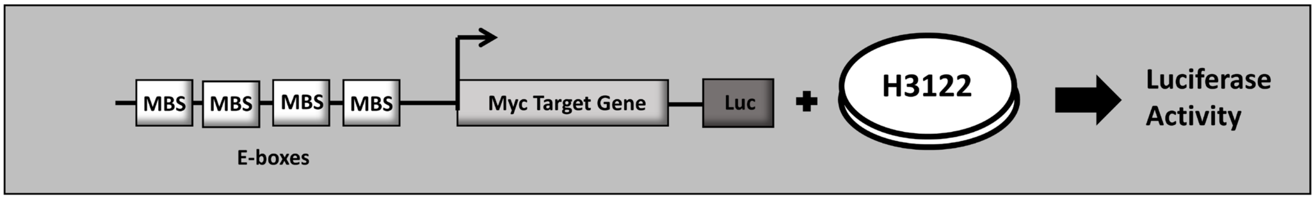

Supplementary Figure 9: MBS-Luc promoter reporter luciferase construct containing four c-MYC binding sites (MBS) and promoter of a known c-MYC target gene (*CDK4*).

Supplementary Table 1A: H2228 genes depleted in crizotinib
For Supplementary Table 1A, please see the attached file

Supplementary Table 1B: H3122 genes depleted in crizotinib
For Supplementary Table 1B, please see the attached file
